# Genetic Impact on Clinical Features in Parkinson's Disease: A Study on *SNCA*-rs11931074

**DOI:** 10.1155/2018/2754541

**Published:** 2018-12-03

**Authors:** Li Shu, Dongxiao Liang, Hongxu Pan, Qian Xu, Jifeng Guo, Qiying Sun, Beisha Tang, Xinxiang Yan

**Affiliations:** ^1^Department of Neurology, Xiangya Hospital, Central South University, Changsha, Hunan 410008, China; ^2^National Clinical Research Center for Geriatric Disorders, Changsha, Hunan 410078, China; ^3^Key Laboratory of Hunan Province in Neurodegenerative Disorders, Central South University, Changsha, Hunan 410008, China; ^4^Center for Medical Genetics, Central South University, Changsha, Hunan 410008, China; ^5^Parkinson's Disease Center of Beijing Institute for Brain Disorders, Beijing 100069, China; ^6^Department of Geriatrics, Xiangya Hospital, Central South University, Changsha, Hunan 410008, China

## Abstract

*SNCA*-rs11931074 had been demonstrated to be strongly correlated with PD risk. However, there was lack of comprehensive analysis of *SNCA*-rs11931074-related clinical features which may help explain clinical heterogeneity of PD. In our study, we performed association analyses on the relationship between *SNCA*-rs11931074 and motor symptoms, nonmotor symptoms, and comorbidities in PD. 611 rs11931074 carriers and 113 rs11931074 noncarriers were enrolled. In the clinical phenotype analyses, the Unified Parkinson's Disease Rating Scale part II (UPDRS II) and part III (UPDRS III) scores of rs11931074 carriers were lower than those of noncarriers (SC: −0.083, *p*=0.035; SC: −0.140, *p* ≤ 0.001). The Charlson Comorbidity Index (CCI) score of carriers was lower than that of noncarriers (SC: −0.097, *p*=0.009). No significant statistical differences were found between the variant and other clinical features such as motor complications and nonmotor symptoms. The *SNCA*-rs11931074 carriers may present with more benign clinical profiles than noncarriers with less severe motor symptoms and comorbidity burden.

## 1. Introduction

Parkinson's disease (PD) is a common neurodegenerative disease characterized by four major motor symptoms: bradykinesia, resting tremor, rigidity, and postural difficulty. Nonmotor symptoms such as cognitive dysfunction, depression, and loss of olfaction are also common clinical presentations of PD [1]. The clinical heterogeneity of PD has been one of the major focuses in PD researches [2–5]. Multiple studies have attributed the clinical heterogeneity of PD to genetic factor. For example, PD patients carrying *GBA* L444P, c.84insG, V394L, or 370Rec, etc. were more likely to have severe motor symptoms and nonmotor symptoms such as cognitive and psychiatric symptoms than idiopathic PD patients [6–8]. *LRRK2* G2385R or R1628P carriers tended to have milder nonmotor symptoms than idiopathic PD patients [9].

*SNCA* was the first gene found in monogenic PD which encoded *α*-synuclein, the major component of pathogenic hallmark Lewy body in PD [10]. rs11931074 in *SNCA* was discovered by genome-wide association studies (GWAS) and had been proven by meta-analysis to be strongly correlated with PD risk [11, 12].

Although there were studies exploring the relationship between *SNCA*-rs11931074 and PD clinical phenotypes, the samples were not large enough to reach a convincible result and the clinical features included in the research studies were not comprehensive. By far, studies had demonstrated that *SNCA*-rs11931074 was associated with age at onset, hyposmia, and REM sleep behavior disorder (RBD) in PD [13–15]. In our study, we made a comprehensive analysis on the relationship between *SNCA*-rs11931074 and clinical features including motor and nonmotor symptoms and comorbidities in PD.

## 2. Methods

### 2.1. Study Population

We randomly selected 724 PD patients from Xiangya Hospital of Center South University in Changsha between 2003 and 2018. All patients were diagnosed by ≥2 experienced neurologists, based on the United Kingdom PD Society Brain Bank Criteria [16]. Patients with a family history of PD were excluded from the study. The genotyping of rs11931074 in this cohort was conducted in article by Guo et al. [17]. Out of 724 patients, there were 611 variant carriers (TT 236 + GT 375) and 113 noncarriers (GG 113). The study was approved by the ethics committee of Central South University and conducted in accordance with the Declaration of Helsinki. All patients provided informed consents before entering the study.

### 2.2. Clinical Assessment

All patients' demographics including age, gender, and clinical characteristics were collected by trained examiners. Mentation, behavior, and mood were assessed by UPDRS I. Activities of daily life were assessed by the UPDRS part II. Motor symptoms were evaluated by UPDRS III, and the disease stage was evaluated by H-Y. We also recorded the medication and calculated LEDD. Cognitive functions were evaluated by MMSE. The score lower than 26 points indicated a cognitive impairment [18]. WO was one of the motor complications with the manifestation of decline of benefit from each dose of levodopa. We evaluated WO by WOQ-9 [19]. The WOQ-9 was used to define the presence of WO phenomenon as the presence of at least one symptom with improvement after the next dose of antiparkinsonism medication [20]. CCI was widely used to assess the common comorbidity conditions [21].

### 2.3. Statistical Analysis

The numerical variable was presented as mean and standard deviation (*X* ± *D*). We used a linear regression model to compare numerical scores after adjusting for age and gender. Numerical variables included UPDRS I score, UPDRS II score, UPDRS III score, H-Y Scale, and LEDD and CCI score. *p* < 0.05 was considered statistically significant. A standardized coefficient (SC) was used to analyze the influence of an independent variable on the dependent variable in the linear regression model. We used the absolute value of SC to measure the influence of the variant on clinical symptoms.

The categorical variable was presented as number of carriers or noncarriers and its relevant frequency separately. A binary logistic regression model was used to compare categorical variables after adjusting for age and gender. Categorical variables contained the WO phenomenon assessed by WOQ-9 and cognitive impairment assessed by the MMSE. *p* < 0.05 was considered of statistical significance. We used odds ratio (OR) to evaluate the risk of each variable in the binary logistic regression model. An OR value of >1 was considered as a risk factor. 95% confidence interval (CI) was estimated.

All statistical analyses were performed using SPSS software (version 22.0; SPSS Inc.). The expression quantitative trait loci (eQTL) of the rs11931074 were found by searching the Braineac database.

## 3. Results

The demographic data and clinical characteristics of PD patients in the carrier group and noncarrier group are shown in Table 1.

### 3.1. Motor Symptoms and Complications

After adjustment for age and gender, linear regression analyses showed that the Unified Parkinson's Disease Rating Scale part II (UPDRS II) and part III (UPDRS III) scores were lower in rs11931074 carriers than those in noncarriers (SC: −0.083, *p*=0.035; SC: −0.140, *p* ≤ 0.001). There was no statistical significance in the Unified Parkinson's Disease Rating Scale part I (UPDRS I) between carrier group and noncarrier group (SC: −0.049, *p*=0.226). There was no statistical significance between the variant and disease stage assessed by Hoehn and Yahr Scale (H-Y) (SC: −0.030, *p*=0.487). No statistical difference was observed in the levodopa equivalent daily dose (LEDD) between the two groups (SC: −0.060, *p*=0.164). The frequency of wearing-off phenomenon (WO) measured by 9-item wearing-off questionnaire (WOQ-9) in carriers was similar to noncarriers (OR: 1.331, 95% CI = 0.578−3.068, *p*=0.520).

### 3.2. Cognitive Dysfunctions and Comorbidities

The Mini-Mental State Examination (MMSE) score was similar between the carrier group and noncarrier group (OR: 0.824, 95% CI = 0.386−1.761, *p*=0.618). For comorbidities of PD, the Charlson Comorbidity Index (CCI) score in carriers was lower than noncarriers (SC: −0.097, *p*=0.009).

### 3.3. eQTL Analysis

In the Braineac database, we found some eQTLs of rs11931074 such as *SNCA* gene (Supplementary Table 1).

## 4. Discussion

Our analysis is a most comprehensive analysis on the relationship between *SNCA-*rs11931074 and clinical characteristics in PD. We included complete demographic data, data of rating scales of motor and nonmotor symptoms, and comorbidities of PD and conducted analysis with by far the largest sample in Chinese populations.

In previous research studies, variants in *SNCA* had been proved to be associated with a series of clinical features which will help explain the clinical heterogeneity of PD. For instance, rs894278 of *SNCA* was related to RBD in PD [14]. rs356182 carriers of PD patients were likely to manifest a tremor-predominant motor symptom [22]. rs11931074 was proved to be strongly associated with PD risk, and there were studies reporting its specific clinical features such as hyposmia and RBD as stated above [12–14].

In our analysis, we observed milder motor symptoms in *SNCA*-rs11931074 carriers in PD patients reflected by UPDRS part II and part III scores than noncarriers. A lower CCI was also found in PD patients carrying *SNCA*-rs11931074 than noncarriers, which may indicate that carriers of the variant presented with a better clinical profile than noncarriers in PD. In that case, *SNCA*-rs11931074 may add to variants with specific clinical profiles in PD and contribute to classifications of PD subtypes. It can also provide support for clinical interventions of the specific features of mutation carriers and guide doctors to conduct symptomatic treatment precisely.

The mechanisms underlying the genotype-phenotype correlations of *SNCA*-rs11931074 may be that the variant could influence the expression of *SNCA* and the following level of *α*-synuclein protein by our finding in eQTL analysis. The protein is a major component of Lewy body which aggregates in dopamine neurons and causes degeneration of dopamine neurons. The dysfunctions of dopamine neurons will lead a series of clinical manifestations especially motor symptoms [23].

There were some inevitable limitations in this study. First, our analysis was a cross-sectional study. A prospective of asymptomatic carriers is needed to reach a more convincing result and provide evidence for genetic counseling. Second, although our sample was the largest sample about clinical features of *SNCA*-rs11931074 in PD, the sample was still not large enough to conduct analyses on the clinical phenotypes of heterozygous or homozygous mutation separately. Third, the interactions of different variants may act as potential biases. Last but not least, as nonmotor symptoms (NMSs) were also vital clinical symptoms of PD, future research studies should pay attention to assess NMSs using specific rating scales such as nonmotor symptom scale (NMS) [24].

## 5. Conclusion

In conclusion, we found that the *SNCA*-rs11931074 carriers may present with more benign clinical profiles than noncarriers.

## Figures and Tables

**Table 1:** Demographic data and clinical characteristics of carrier and noncarrier group of *SNCA-*rs11931074 in PD patients.

	Carriers		Noncarriers		*p*	SC/OR (95% CI)
	*N*	*X* ± *S*/*N* ^*∗*^ (*F*)	*N*	*X* ± *S*/*N* ^*∗*^ (*F*)		
Age of enrollment	611	62.52 ± 12.88	113	64.80 ± 12.11	NA	NA
Age of onset	611	57.91 ± 13.44	113	60.02 ± 12.35	NA	NA
Disease course	611	4.61 ± 4.42	113	4.78 ± 3.97	0.700	0.446 (−1.046 to 0.703)^#^
Gender, male	611	352 (57.6%)	113	64 (56.6%)	NA	NA
UPDRS I	522	1.76 ± 1.95	91	2.03 ± 2.04	0.226	−0.049
UPDRS II	522	12.41 ± 7.13	91	14.26 ± 6.90	**0.035**	−0.083
UPDRS III	553	27.42 ± 15.77	97	34.18 ± 16.53	**≤0.001**	−0.140
H-Y	452	2.26 ± 0.95	80	2.38 ± 0.89	0.487	−0.030
LEDD	469	546.78 ± 300.44	83	594.09 ± 115.41	0.164	−0.060
CCI	611	0.10 ± 0.37	113	0.21 ± 0.47	**0.009**	−0.097
Cognitive impairment	247	63 (25.5%)	39	12 (30.8%)	0.618	0.824 (0.386–1.761)
WO	403	51 (12.7%)	71	7 (9.9%)	0.502	1.331 (0.578–3.068)

UPDRS I, II, and III: Unified Parkinson's Disease Rating Scale parts I, II, and III; H-Y: Hoehn and Yahr Scale; LEDD: levodopa equivalent daily dose; CCI: Charlson Comorbidity Index score; WO: wearing-off phenomenon evaluated by the 9-item wearing-off questionnaire (WOQ-9); *N*: the total number of carriers and noncarriers of *SNCA-*rs11931074 in PD patients; SC: standardized coefficient; OR: odds ratio; CI: confidence interval; NA: not available. ^#^Standard error (95% CI). Cognitive impairment was assessed by Mini-Mental State Examination (MMSE). Numerical variable was presented as mean and standard deviation (*X* ± *S*); categorical variable was presented as the number of carriers or noncarriers and its relevant frequency separately [*N* ^*∗*^ (*F*)]. *p* value <0.05 was considered statistically significant and is shown in bold.

## Data Availability

The data used to support the findings of this study are included within the article and the supplementary file.

## Supplementary Materials

Supplementary Materials Table S1: results of eQTLs from the Braineac database.
