# Supplementary material for: Genetic Impact on Clinical Features in Parkinson's Disease: A Study on SNCA-rs11931074
Source: Parkinsons Dis. 2018 Dec 3;2018:2754541. doi: 10.1155/2018/2754541 (PMC6304873; doi:10.1155/2018/2754541)
Supplement: Supplementary Materials — Table S1: results of eQTLs from the Braineac database. [file 2754541.f1.pdf]

Supplementary Table 1. Results of eQTLs from Braineac database.

geneSymbol	marker	rsid	exprID	chr	start	stop	aveALL	CRBL	FCTX	HIPP	MEDU	OCTX	PUTM	SNIG	TCTX	THAL	WHMT
MMRN1	chr4:90639515	rs11931074	2735759	chr4	9.1E+07	9.1E+07	**9.50E-03**	**2.80E-03**	5.30E-02	6.60E-01	7.00E-01	**1.80E-02**	5.60E-01	6.80E-01	3.00E-01	**2.50E-04**	4.20E-01
FAM190A	chr4:90639515	rs11931074	2735862	chr4	9.1E+07	9.3E+07	**3.30E-02**	4.70E-01	7.60E-01	5.50E-01	1.50E-01	2.90E-01	4.60E-01	6.60E-01	1.70E-01	1.70E-01	**7.70E-04**
MMRN1	chr4:90639515	rs11931074	2735775	chr4	9.1E+07	9.1E+07	**4.90E-02**	9.00E-02	1.40E-01	4.00E-01	3.00E-01	3.20E-01	2.30E-01	6.20E-01	5.70E-02	**1.50E-03**	4.30E-01
MMRN1	chr4:90639515	rs11931074	2735782	chr4	9.1E+07	9.1E+07	**2.80E-02**	1.90E-01	3.70E-01	6.90E-01	9.00E-01	9.00E-01	3.90E-01	9.40E-02	5.50E-02	**1.60E-03**	3.10E-01
FAM13A	chr4:90639515	rs11931074	2777589	chr4	9E+07	9E+07	6.30E-01	7.50E-01	1.20E-01	6.60E-01	**2.00E-03**	6.20E-01	4.70E-01	6.30E-01	8.20E-01	1.70E-01	1.10E-01
MMRN1	chr4:90639515	rs11931074	2735768	chr4	9.1E+07	9.1E+07	2.30E-01	6.50E-01	5.90E-01	7.40E-01	1.20E-01	**8.50E-03**	5.30E-01	2.30E-01	8.40E-01	1.40E-01	**2.40E-03**
FAM13A	chr4:90639515	rs11931074	2777595	chr4	9E+07	9E+07	2.10E-01	5.30E-01	7.70E-01	7.40E-01	**3.60E-03**	7.90E-01	9.60E-01	2.30E-01	9.70E-01	4.00E-01	8.00E-01
FAM13A	chr4:90639515	rs11931074	2777593	chr4	9E+07	9E+07	9.90E-01	8.90E-01	6.20E-01	8.90E-01	2.30E-01	6.20E-01	7.80E-01	1.30E-01	9.90E-01	2.80E-01	**3.80E-03**
MMRN1	chr4:90639515	rs11931074	2735764	chr4	9.1E+07	9.1E+07	**3.20E-02**	6.70E-02	5.80E-02	9.10E-01	5.70E-01	**4.00E-03**	6.90E-01	8.10E-01	1.60E-01	9.10E-02	3.60E-01
SNCA	chr4:90639515	rs11931074	2777715	chr4	9.1E+07	9.1E+07	8.30E-02	9.60E-02	8.90E-01	6.80E-01	4.50E-01	1.00E+00	5.20E-01	6.10E-01	**2.30E-02**	4.00E-01	**5.50E-03**

Abbreviations: Ave All, average across all ten regions; CRBL, cerebellum; FCTX, frontal cortex; HIPP, hippocampus; MEDU, medulla; OCTX, occipital cortex; PUTM, putamen; SNIG, substantia nigra; TCTX, temporal cortex; THAL, thalamus; WHMT, white matter. The bold text represents statistically significant results.
